# Intersection management for autonomous vehicles with vehicle-to-infrastructure communication

**DOI:** 10.1371/journal.pone.0235644

**Published:** 2020-07-02

**Authors:** Yuying Li, Qipeng Liu

**Affiliations:** Institute of Complexity Science, Qingdao University, Qingdao, China; Tongii University, CHINA

## Abstract

This paper proposes an intersection management strategy for autonomous vehicles under the vehicle-to-infrastructure circumstance. All vehicles are supposed to be fully autonomous and can communicate with the intersection management unit to check the traffic situation. Priority of passing the intersection is decided by a static conflict matrix which represents the potential conflict between lanes of different directions and a dynamic information list which could capture the real-time occupation of each lane in the intersection. Compared with the existing approaches in the literature, the intersection management unit in our strategy is more like a database rather than a computational center, and therefore, requires less computational resource and more likely satisfies the real-time requirement in heavy traffic situations. Simulations are conducted using SUMO (Simulation of Urban MObility), in which the proposed strategy is compared with both fixed and adaptive traffic light methods. The results indicate that the proposed strategy could significantly reduce the average time delay caused by the intersection and the corresponding variance, which shows the efficiency and fairness of the proposed strategy in intersection management.

## Introduction

The intersection plays an important role in the traffic network, which is also one of the main causes of traffic accidents. Based on the statistical data, about 40 percent of the crashes that occurred in the United States in 2008 were intersection-related [1]. Crashes near intersections might also lead to serious traffic jams on multiple roads, which apparently waste time and money of drivers and also cause unnecessary air pollution. It is also reported that about 96 percent of the intersection-related crashes had critical reasons attributed to drivers, such as inadequate surveillance, false assumption of other’s action, and turned with obstructed view [1, 2].

Autonomous vehicles (AVs) as a promising solution to traffic accidents have attracted much attention recently. As shown in its official blog, Waymo, an autonomous driving technology development company originated from Google, has already started a commercial self-driving taxi service in Phoenix, Arizona in 2018 [3]. An optimistic prediction is that AVs will publicly available in the next decade, and thus traffic problems related to autonomous vehicles are also being extensively investigated [4, 5]. Current intersection management (IM) strategies, such as the traditional phase-fixed traffic light and other more advanced adaptive methods, are designed exclusively for human drivers. With the rapid development of AVs, new IM strategies taking into account of AVs should be designed. As vehicle-to-infrastructure (V2I) communication is a basic requirement in the future intelligent transportation system (ITS), it should also be used in IM [6].

The intersection management problem dealing with autonomous vehicles and V2I has been a hot topic in the field of ITS in the past decades. For example, Dresner and Stone established a multi-agent intersection coordination and control unit based on intersection resource reservation technology in the V2I environment [7]. When the vehicle enters the control area of the intersection, it will make a pass request to the control unit, and then a decision will be made based on the policy of “First Come First Serve” (FCFS). Similarly, Li et al. considered to divide the intersection into a mesh of *n*-by-*n* tiles, where *n* is termed granularity and reflects the tile density of the intersection mesh [8]. The IM unit sends an approval or rejection message to the vehicle with a designated acceleration or deceleration rate that will result in no conflict with existing reservations. Based on Dresner’s work, Huang et al. proposed an approach considering more features to better represent the real-world driving environment [9]. Chouhan and Banda proposed an intuitive heuristic method to solve the space-time conflicts during the driving of the vehicle, such that the vehicle can go through the intersection safely [10].

Beside the above heuristic-based approaches, the intersection management problems can also be solved by optimization with different objective functions. For example, Lu and Kim proposed a mixed integer programming based intersection coordination algorithm to generate the fastest trajectory for each vehicle approaching the intersection [11]. Cruz-Piris et al. used genetic algorithms to optimize the vehicle arrival rate and developed a cellular automaton simulator to simulate a variety of traffic scenes [12]. Zhao et al. solved the multi-objective optimization problem under the multi-constraint condition of connected vehicles and automated vehicles at non-signal intersections [13]. Zhang et al. studied how to minimize the energy consumption of connected and autonomous vehicles at urban intersections as well as meet the requirement of throughput maximization [14]. Creemers et al. proposed an IM algorithm based on model predictive control (MPC) to reduce the average delay time of vehicles [15]. Wu et al. proposed the decentralized coordination learning strategy to optimize control policy [16].

Most of the IM strategies in the literature require the IM unit to possess high computing power for solving optimization problems or calculating proper acceleration and deceleration rate for the approaching vehicles in real time. In this paper we propose an IM strategy that relax this requirement. In our strategy, the IM unit is more like a database rather than a computational center, and therefore, requires less computational resource and more likely satisfies the real-time requirement in heavy traffic situations.

The contributions of this paper are as follows. We propose an IM strategy taking the vehicle-to-infrastructure communication into account. The IM unit maintains information reflecting the current occupation of the intersection. The arriving vehicle could retrieve the traffic information once it enters the communication range of the IM unit, and then accordingly adjusts its speed to make sure entering the intersection no earlier than the safe arriving time. All the heavy computational tasks are performed on the vehicle side, which greatly reduces the pressure on the IM unit. Simulations are conducted using SUMO (Simulation of Urban MObility) and the results show that the proposed strategy could significantly reduce the average delay time caused by the intersection, compared with the widely-used traffic light methods.

## Problem formulation

### Intersection environment settings

**Fig 1:**
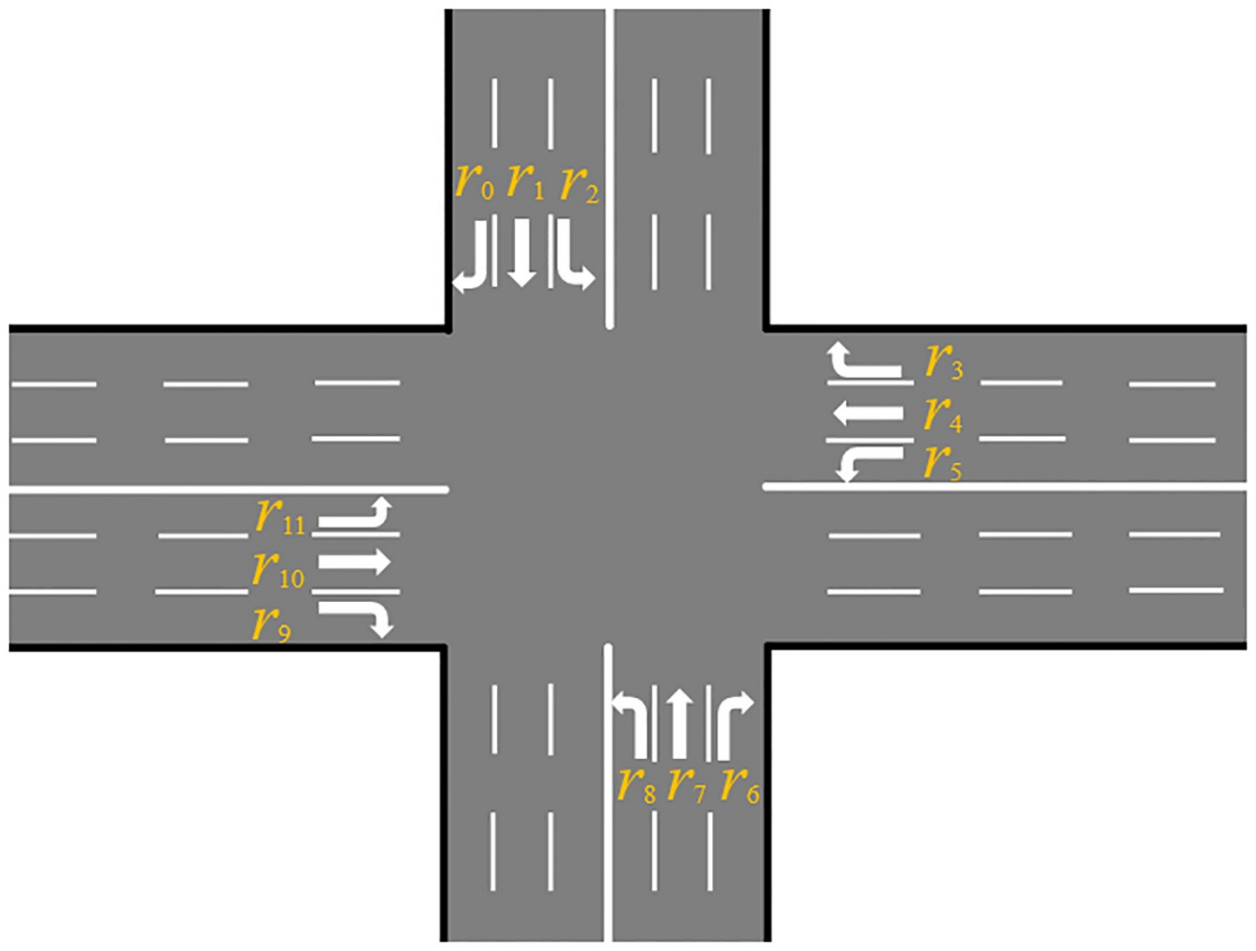
The intersection environment considered in this paper. It is a widely-seen crossroad with three incoming and outgoing lanes in each direction.

There exist various shapes of intersections. For the sake of easy description, we focus on the widely seen four-way intersection (or called crossroad) shown in Fig 1. Our strategy is supposed to be easily extended to many other shapes of intersections.

There are 12 incoming lanes. Each lane has a unique id *r*_*i*_ (*i* = 0, 1, ⋯, 11), numbered in clockwise starting from the top-most. The simplicity of this intersection is that each lane has only one outgoing direction. That is, the route of the vehicle in the intersection area can be uniquely represented by the corresponding lane, which makes the analysis much easier.

### Assumptions

Before designing the IM strategy, we need to be clear about the traffic situation we are facing. Suppose that the following assumptions are always hold under our circumstance:

1. All overtaking and lane changing have already been completed, and therefore, vehicles driving on a lane must follow the direction of the lane.
2. All vehicles are fully autonomous, which could drive in a given lane at a given speed. Other traffic units such as pedestrians and emergency vehicles are not considered.
3. All vehicles can perfectly communicate with the IM unit once they get into the communication range. That is, there is no time delay, data packet loss, or malicious data injection during the communication. The technical details realizing this V2I communication are beyond the scope of this paper.
4. All speed and time estimations performed by AVs are accurate enough. These estimations are the basis for subsequent decisions made by AVs.

## Intersection management based on V2I communication

### Conflict matrix

We say two routes are conflict if they intersect with each other at some point inside the traffic intersection area. In the situation shown in Fig 1, routes of vehicle can be reflected by lanes the vehicles occupy. Therefore, we can also say two lanes are conflict or not. From Fig 1 we can figure out whether two lanes conflict with each other and then we can establish a conflict matrix for all lanes. The rows and columns represent the lanes and if two lanes conflict with each other, the element at the corresponding row and column is one; otherwise, zero. The entire conflict matrix is shown in Table 1.

**Table 1:** Conflict matrix.

	*r*_0_	*r*_1_	*r*_2_	*r*_3_	*r*_4_	*r*_5_	*r*_6_	*r*_7_	*r*_8_	*r*_9_	*r*_10_	*r*_11_
*r*_0_	1	0	0	0	0	0	0	0	0	0	0	0
*r*_1_	0	1	0	0	1	0	0	0	1	0	1	1
*r*_2_	0	0	1	0	1	1	0	1	0	0	0	1
*r*_3_	0	0	0	1	0	0	0	0	0	0	0	0
*r*_4_	0	1	1	0	1	0	0	1	0	0	0	1
*r*_5_	0	0	1	0	0	1	0	1	1	0	1	0
*r*_6_	0	0	0	0	0	0	1	0	0	0	0	0
*r*_7_	0	0	1	0	1	1	0	1	0	0	1	0
*r*_8_	0	1	0	0	0	1	0	0	1	0	1	1
*r*_9_	0	0	0	0	0	0	0	0	0	1	0	0
*r*_10_	0	1	0	0	0	1	0	1	1	0	1	0
*r*_11_	0	1	1	0	1	0	0	0	1	0	0	1

This conflict matrix is static for a given traffic intersection and maintained by the IM unit. It represents the potential conflict among vehicles driving on any lanes. If one vehicle approaches from a lane without any potential conflict (e.g., lanes for right turn), the vehicle can safely pass the intersection at desired speed. Otherwise, one should further check the actual occupation of the intersection in real time, which could be reflected by a dynamic information list.

### Information list

In our design, an information list contains a list of vehicles with associated information:

- Vehicle ID: *i* ∈ {0, 1, 2, ⋯}
- Vehicle route ID: *route*_*i*_ ∈ {*r*_0_, *r*_1_, *r*_2_, ⋯, *r*_11_}
- The time that the vehicle leaving the intersection: *t*_*i*_ ∈ [0, ∞)

This information list is also maintained by the IM unit.

Next we will describe how the information list is updated and how the whole IM system works.

### Traffic control strategy

The control strategy is shown as follows:

(1) Initial state: Information list is empty.
(2) Once a vehicle gets into the communication range of the IM unit (may be hundreds meters away), the basic information of the vehicle is sent to the IM unit, including its vehicle ID, say *i*, and its route ID, *route*_*i*_. The IM unit checks the conflict matrix first. If there exists no potential conflict at all (this happens to right-turning vehicles), the IM unit will send a *NULL* signal to the vehicle indicating safely passing the intersection and then turn to step (4). Otherwise, find out the potential conflict routes of *route*_*i*_. Take *route*_*i*_ = *r*_1_ for example, the potential conflict routes are *r*_1_, *r*_4_, *r*_8_, *r*_10_, and *r*_11_.
(3) Search information list and find out all vehicles currently on the potential conflict routes. Send the corresponding maximum leaving time *t*_*max*_ back to vehicle *i*. If there is no vehicle on the conflict route, set *t*_*max*_ to be *NULL*.
(4) Vehicle *i* adjusts its speed according to the feedback information from the IM unit. Especially it should make sure to arrive at the intersection later than *t*_*max*_ if the the feedback signal is not *NULL*. Note that AVs could adopt various speed adjustment methods. The guide line is that the vehicle should pass the intersection as soon as possible but later than *t*_*max*_. A safe time gap may be also token into account. Finally, the vehicle sends its estimate time of leaving the intersection, *t*_*i*_, to the IM unit.
(5) The IM unit updates the information list by adding *i*, *route*_*i*_, and *t*_*i*_. Any vehicle leaving the intersection should send a signal to the IM unit, such that the IM unit can delete its information from the list.
(6) Repeat steps (2) to (5).

**Fig 2:**
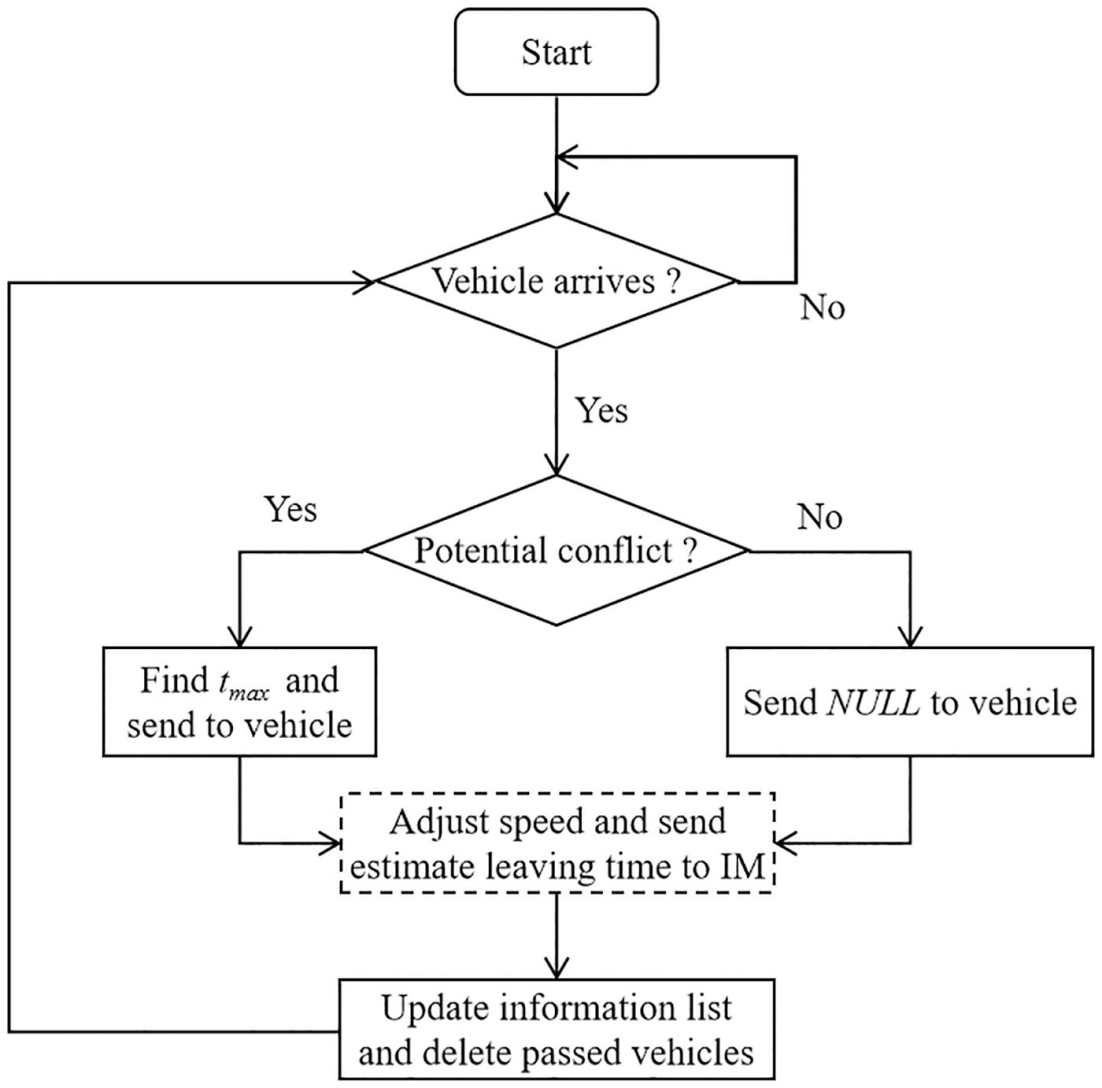
Flowchart of the proposed IM strategy. The work of checking table is done on the IM side while the heavy computing work is done on the vehicle side shown in the dashed box.

The flowchart of the above steps is shown in Fig 2, in which the step inside the dashed box is performed on the vehicle side.

The pseudo code of the algorithm performed by IM unit is given in Algorithm 1:

**Algorithm 1**: Searching maximum leaving time on conflict lanes

 **Input**: Conflict matrix *M*, information list *L*, the approaching vehicle ID *i*, and its route ID *route*_*i*_

 **Output**: Maximum leaving time *t*_*max*_ on *i*’s conflict lanes

1: **if** *route*_*i*_ has no conflict lane in *M* **then**

2:  *t*_*max*_ = *NULL*

3: **else**

4:  **foreach** conflict lane *r*_*k*_ in *M* **do**

5:   Get leaving time *t*_*k*_ on lane *r*_*k*_ from *L*

6:  **end**

7:  *t*_*max*_ = max{*t*_*k*_}

8: **end**

In our IM strategy, the IM unit mainly performs table searching work and tells the vehicle the earliest safe arriving time. The vehicle is responsible for adjusting speed and estimating the time to pass the intersection. Compared with the approaches in the literature where the IM unit is supposed to compute the reduced speed through reservation scheme or optimization method, our strategy requires less computational power on the IM unit side and is more likely to deal with heavy traffic situations.

## Simulations in SUMO

In order to verify the efficiency of the proposed IM strategy, we conduct simulations using an open-source traffic system simulation tool—SUMO (Simulation of Urban MObility) and its extension Plexe [17, 18]. We consider the intersection environment shown in Fig 1. The parameters in simulations are listed as follows:

- The distance from starting point of each road to the stop line before the intersection is 500 meters.
- The distance from stop line to the center of intersection is 15 meters.
- Suppose that the IM unit is located at the center of the intersection and the communication range of the IM unit is 200 meters.
- The normal speed of vehicle is 60 km/h, that is about 16.67 m/s.
- Vehicle length is 4 meters.

Other parameters are set to the default values provided by SUMO.

In our IM strategy, we do not specify how the vehicle adjusts speed. Any method is acceptable as long as the vehicle arrives at the intersection after *t*_*max*_ + *t*_*δ*_, where *t*_*max*_ is the maximum leaving time of other vehicles on the conflict lanes and *t*_*δ*_ = 1*s* is the additional safe gap time. In the simulation, we use a simplified speed adjustment method: once the vehicle receives *t*_*max*_, if speed reducing is needed, it adjusts speed to *D*/(*t*_*max*_ + *t*_*δ*_ − *t*_*c*_), where *D* is the distance from the current position of the vehicle to the stop line and *t*_*c*_ is the current time.

**Fig 3:**
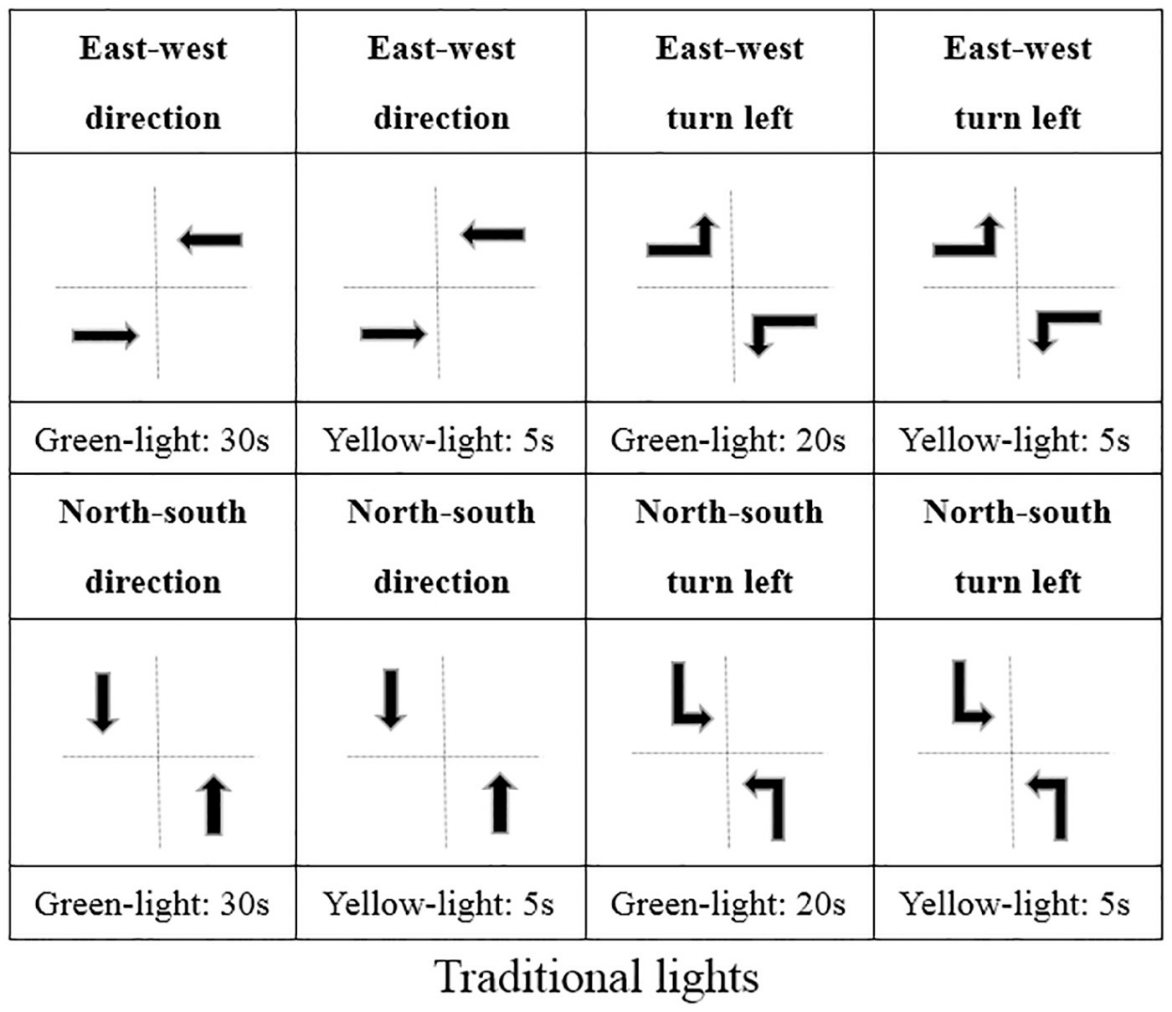
The phase transformation diagram of phase-fixed lights.

For the purpose of comparison, we also examine the traditional phase-fixed traffic light and the adaptive traffic light. The traditional traffic light uses a three-phase signal timing scheme, with a total signal cycle of 120 seconds, in which the green light for going straight in each direction is 30 seconds, the yellow light 5 seconds, the green light for turning left 20 seconds, the yellow light 5 seconds, and then the red light for the rest time. A phase transformation diagram is shown in Fig 3.

**Fig 4:**
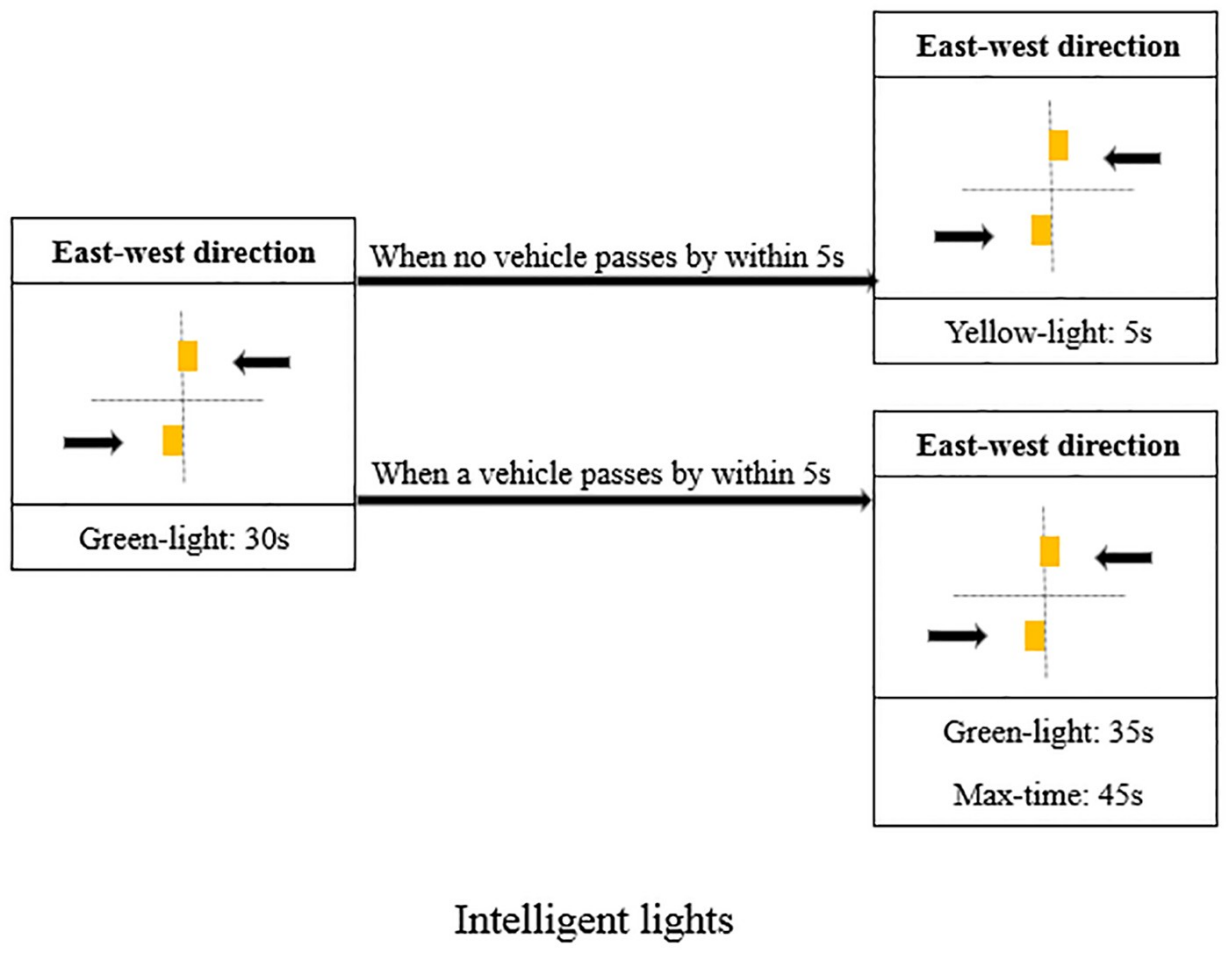
The phase transformation diagram of adaptive lights.

The adaptive traffic light monitors the number of vehicles in real time by inductive loop detectors which are deployed 15 meters before the intersection stop line. If an approaching vehicle is detected and meanwhile the traffic light is green, then the traffic light will automatically extend the green phase for 5 seconds. The maximum duration of green light is 45 seconds. If no vehicle is detected within 5 seconds before the end of green light phase, the light changes to the next phase. A phase transformation diagram (only considering the east-west direction) is shown in Fig 4.

All of the simulation codes are shared at the following Github repository: https://github.com/TianzhenLi/Intelligent-Intersection. The results can be reproduced following the instruction. The final statistical data files are also provided which could generate exact the same results as in our paper.

### Simulation 1: Same traffic density on each lane

The first simulation considers same traffic density on each lane, in which vehicles are generated every 6 seconds with probability of 0.3 at the starting point of each lane.

To quantify the efficiency of IM strategies, we use two indicators: the average delay time of all vehicles, $\bar{d}$, and the corresponding variance, *σ*^2^. From the statistical data file generated by SUMO, we extract the delay time of each vehicle *d*_*i*_, *i* ∈ {1, 2, …, *N*}, and calculate $\bar{d}$ and *σ*^2^ as follows:

$$\begin{array}{c} \bar{d}=\frac{\Sigma_{i=1}^{N}d_{i}}{N},\;\;\;\sigma^{2}=\frac{\Sigma_{i=1}^{N}{\left(d_{i}-\bar{d}\right)}^{2}}{N} \end{array}$$

Intuitively, the average delay time represents the efficiency of the IM strategy and the variance of delay time can tell us how fairness the strategy is. A good strategy should result in both small average delay time and small variance. We can see from Table 2 that the proposed strategy is much better than others, and the adaptive light is slightly better than the traditional phase-fixed light. The underlying reason is that, in our strategy vehicles could communicate with the IM unit and change its speed according to the feedback information concerning the occupation of the intersection. Due to the early adjustment, vehicles could safely go through the intersection without unnecessary stop, and for most of the cases only speed reducing is needed.

**Table 2:** Average time delay and its variance.

	Phase-fixed	Adaptive	Ours
Total number of veh.	2098	2070	2152
Average delay (s)	28.48	20.48	1.75
Variance	967.55	500.93	0.36

### Simulation 2: Unbalanced traffic density

In the above simulation, the traffic densities of all directions are almost the same. Now we consider an intersection which has unbalanced traffic densities in different directions. We set the number of vehicles coming from the north-south road much greater than that from the east-west road. In the simulation, vehicles are generated for every 6 seconds with probability of 0.3 in the north-south direction, while with probability of 0.03 in the east-west direction.

Again we examine the three IM strategies and show the result in Table 3.

**Table 3:** Average time delay and its variance in unbalanced case.

	Phase-fixed	Adaptive	Ours
Total number of veh.	1150	1141	1132
Average delay (s)	29.90	13.42	2.02
Variance	991.90	205.14	0.21

The results show that our proposed strategy is still quite efficient in this unbalanced traffic density situation. Moreover, comparing Tables 2 and 3, we can see that the adaptive traffic light is quite suitable for the unbalanced traffic situation. By adaptive adjustment, the green-light phase for the north-south direction is much longer than the other direction, which correctly reflects the different traffic densities.

### Simulation 3: The influence of traffic density

**Fig 5:**
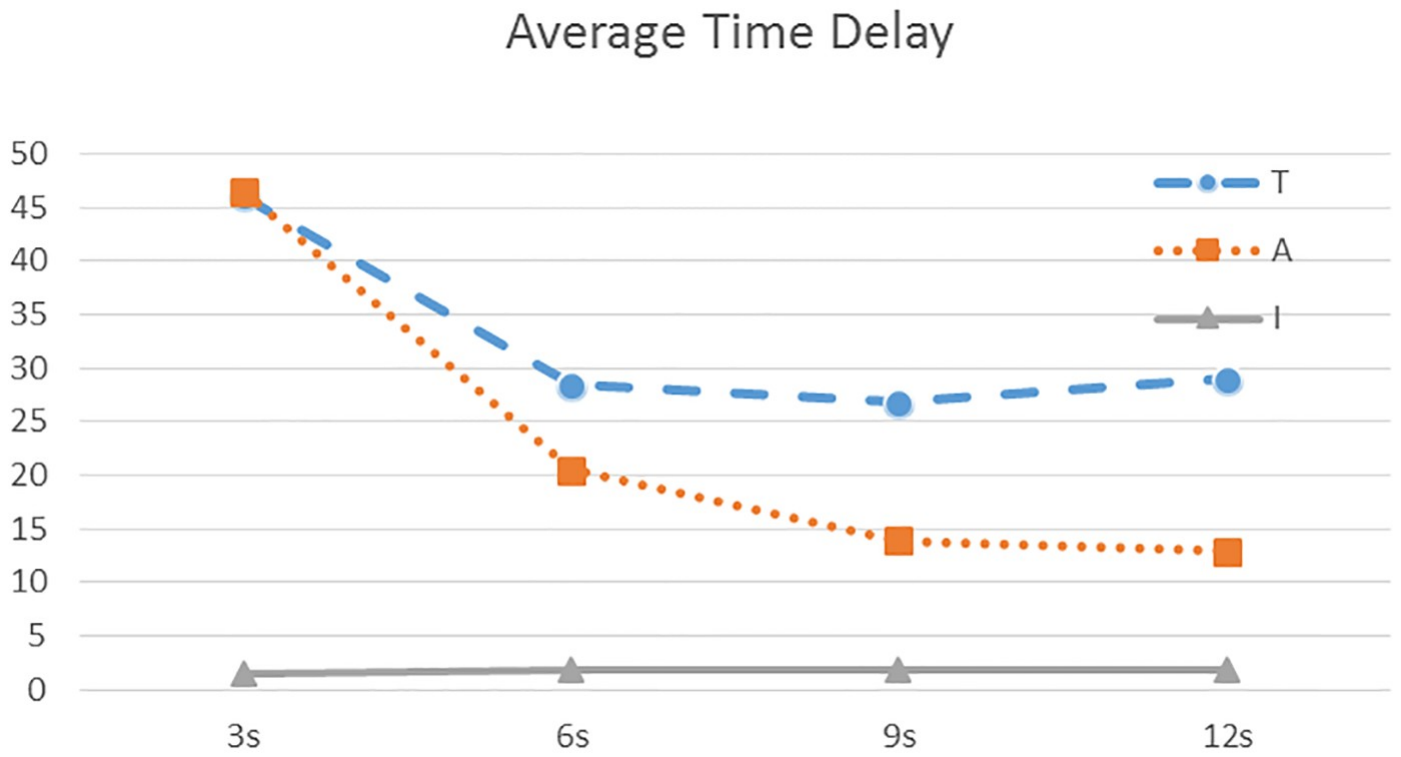
Comparison of average time delay using the three traffic control strategies under different traffic densities. The horizontal axis is the time interval of generating vehicles at the starting point of the road. A small value corresponds to a heavy traffic situation. The line labeled **T** represents the traditional traffic light, **A** for adaptive light, and **I** for the proposed intelligent strategy.

**Fig 6:**
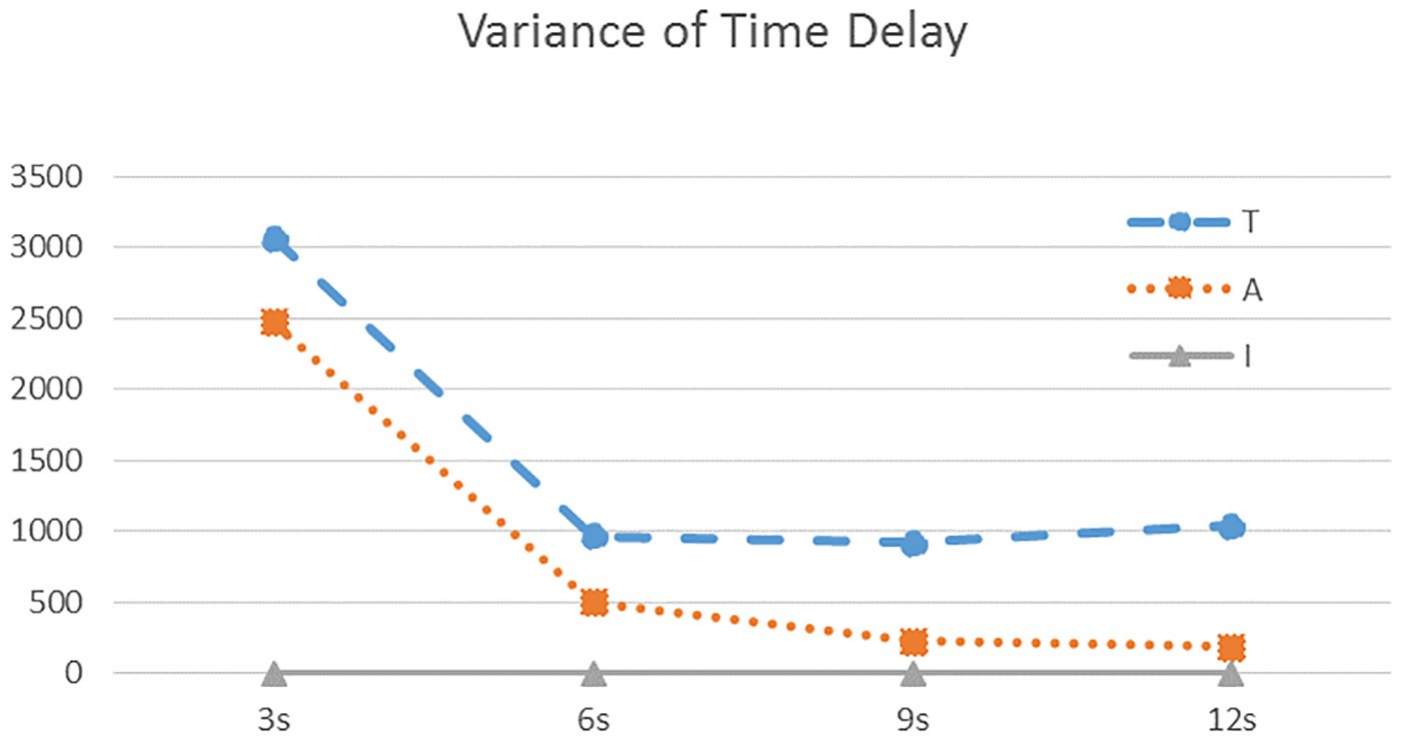
Comparison of variance of time delay using the three IM strategies, under different traffic densities.

In this part, we examine the influence of traffic density on the three IM strategies. The time intervals for generating vehicles are chosen to be 3 seconds, 6 seconds, 9 seconds, and 12 seconds, respectively. The results are shown in Table 4. To have a more intuitive understanding, we also plot the results in Figs 5 and 6.

**Table 4:** Results from different traffic densities.

Interval		Fixed	Adaptive	Ours
3 s	No. of vehicles	4263	4161	4303
	Average delay (s)	46.07	46.54	1.45
	Variance	3066.44	2471.01	0.45
6 s	No. of vehicles	2098	2070	2152
	Average delay (s)	28.48	20.48	1.75
	Variance	967.55	500.93	0.36
9 s	No. of vehicles	1415	1436	1436
	Average delay (s)	26.79	13.87	1.87
	Variance	922.40	228.69	0.36
12 s	No. of vehicles	1051	1045	1042
	Average delay (s)	28.95	12.88	1.86
	Variance	1039.76	191.67	0.37

The following conclusions can be drawn from the simulations:

1. Among the three strategies, ours has the minimum average time delay and variance, and thus, the highest traffic efficiency at the intersection, for both heavy and light traffic situations.
2. For the light traffic situations, adaptive method is apparently better than the traditional traffic light. When there are many vehicles in one direction, appropriately extending the green-light phase for that direction can reduce the unnecessary waiting time and improve the traffic efficiency.
3. As the traffic density increases (i.e., the time interval of generating vehicles decreases from 12 seconds to 3 seconds), the average delay and its variance under the traditional and adaptive light increase rapidly. In extreme cases, a lone queue of vehicles will be established. However, our strategy is almost not influenced by the heavy traffic. We can see from Figs 5 and 6 that the average delay and its variance stay at a low level.
4. For the quite heavy traffic situation (e.g., 3s case), both traditional and adaptive light have poor performance. The adaptive method has no apparent advantage compared with the traditional one.
5. By calculation, compared with adaptive traffic lights, our strategy can reduce the time delay by 90 percent on average, while compared with traditional traffic lights, our strategy can reduce the time delay by 94 percent on average.

## Conclusion and future work

In this paper, we propose an intersection management strategy based on the vehicle-to-infrastructure communication. Unlike the existing approaches in the literature, our strategy removes the heavy computational burden from the center management unit which now only needs to maintain a static conflict matrix and a dynamic information list, and perform the tasks such as storing and searching. Simulations using SUMO show that our method has much higher traffic efficiency and fairness compared with the traditional phase-fixed and adaptive traffic light.

The intersection management strategy proposed in this paper is essentially heuristic. It is probably not the optimal one. A better algorithm might involve solving optimization problems on the vehicle side, if we do not want to add computational burden to the center management unit. Some promising research directions are list as follows:

- Multiple intersections: IM strategy could systematically consider multiple upstream and downstream intersections to improve the overall efficiency of the traffic network.
- Vehicle platooning: With the upcoming vehicle-to-vehicle communication technology, platooning of vehicles with small safety distance is a promising solution to increase the road throughput and reduce energy consumption.
- Coexistence of autonomous vehicles and human-operated vehicles: Autonomous vehicles will not completely replace human-operated vehicles in a short time. In the near future, autonomous and human-operated vehicles will coexist on the road. Therefore, intersection management considering this coexistence is necessary.
- Trajectory optimization: Allowing vehicles to have more freedom to optimize their decisions (e.g., overtaking and changing lanes) near the intersection area might further improve the traffic efficiency.
